# The mitogenome of the bed bug *Cimex lectularius* (Hemiptera: Cimicidae)

**DOI:** 10.1080/23802359.2016.1180268

**Published:** 2016-06-20

**Authors:** Sergios-Orestis Kolokotronis, Jonathan Foox, Jeffrey A. Rosenfeld, Mercer R. Brugler, Darryl Reeves, Joshua B. Benoit, Warren Booth, Grant Robison, Michael Steffen, Zoe Sakas, Subba R. Palli, Coby Schal, Stephen Richards, Apurva Narechania, Richard H. Baker, Louis N. Sorkin, George Amato, Christopher E. Mason, Mark E. Siddall, Rob DeSalle

**Affiliations:** a Department of Biological Sciences, Fordham University, Bronx, NY, USA;; b Sackler Institute for Comparative Genomics and Division of Invertebrate Zoology, American Museum of Natural History, New York, NY, USA;; c Cancer Institute of New Jersey, Rutgers University, New Brunswick, NJ, USA;; d Department of Biological Sciences, NYC College of Technology, City University of New York, Brooklyn, NY, USA;; e Department of Physiology and Biophysics and HRH Prince Alwaleed Bin Talal Bin Abdulaziz Alsaud Institute for Computational Biomedicine, Weill Cornell Medicine, New York, NY, USA;; f Department of Biological Sciences, University of Cincinnati, Cincinnati, OH, USA;; g Department of Biological Sciences, the University of Tulsa, Tulsa, OK, USA;; h Department of Entomology, University of Kentucky, Lexington, KY, USA;; i Department of Entomology and W.M. Keck Center for Behavioral Biology, North Carolina State University, Raleigh, NC, USA;; j Department of Human and Molecular Genetics, Human Genome Sequencing Center, Baylor College of Medicine, Houston, TX, USA

**Keywords:** Arthropods, blood feeding, Hemiptera, pest, true bugs

## Abstract

We report the extraction of a bed bug mitogenome from high-throughput sequencing projects originally focused on the nuclear genome of *Cimex lectularius*. The assembled mitogenome has a similar AT nucleotide composition bias found in other insects. Phylogenetic analysis of all protein-coding genes indicates that *C. lectularius* is clearly a member of a paraphyletic Cimicomorpha clade within the Order Hemiptera.

The common bed bug, *Cimex lectularius* Linnaeus, 1758 (Hemiptera: Cimicidae), has been intimately associated with humans for thousands of years (Panagiotakopulu & Buckland 1999; Booth et al. 2015). It is an obligate ectoparasite that primarily feeds on human blood in a haematophagic lifestyle, but will readily feed on many bird and mammalian species as well (Usinger 1966). The insecticide-susceptible laboratory strain Har-73 (= Harlan) of *C. lectularius* was used for whole-genome shotgun sequencing and sequence assembly *de novo*, performed by two independent research groups: one in New York City, based primarily at the American Museum of Natural History (AMNH) and Weill Cornell Medicine (WCM) (Rosenfeld et al. 2016), and another one at Baylor College of Medicine (BCM) as part of the i5k genome-sequencing initiative (http://www.arthropodgenomes.org/wiki/i5K) (Benoit et al. 2016). Specimens are kept in the AMNH Invertebrate Zoology collection and stored in liquid nitrogen in the Ambrose Monell Cryo Collection (AMCC). Living colonies are maintained by L. N. Sorkin and fed on human blood and an inbred line is maintained by C. Schal and fed on rabbit blood. Purified DNA and RNA samples are also stored in the AMCC and at WCM. Sequence data for the original genome projects can be accessed on GenBank/EMBL/DDBJ under BioProject PRJNA259363 and PRJNA167477. In order to identify orthologous loci and assemble the bed bug mitogenome, we first queried complete mitogenome sequences from related species *Adelphocoris fasciaticollis* (GenBank accession no. NC_023796), *Empoasca vitis* (NC_024838), *Orius sauteri* (NC_024583), *Peirates arcuatus* (NC_024264) and *Triatoma dimidiata* (NC_002609) against the genome assemblies of both genome projects. This did not yield significant hits; therefore, we subsequently queried the abovementioned mitogenome sequences against the high-quality Illumina reads (Illumina Inc., San Diego, CA), which yielded bona fide matches. The mitogenome assembly was annotated using MITOS (Bernt et al. 2013), resulting in 13 protein-coding genes, two rRNA loci and 21 tRNA loci (tRNA-Leu was missing), and a mitogenome size of 15,217 bp. The mitogenome sequence is deposited in GenBank under accession code KU350871. We compared our mitogenome sequence to a subset of related mitogenome sequences (Li et al. 2011) in a maximum likelihood phylogenetic analysis using the 13 encoded proteins in RAxML 8.2.4 (Stamatakis 2014) with the MtArt replacement matrix (Abascal et al. 2007) and empirical residue frequencies, along with among-site rate heterogeneity modeled with the Γ distribution and four discrete rate categories (Yang 1994), through 10 searches starting from random-addition maximum parsimony trees (Figure 1(A)). *Cimex lectularius* was placed as sister to *Orius niger* and *Lygus lineolaris* within the Cimicomorpha clade. This is in agreement with a larger phylogenetic analysis of hemipteran mitochondrial genomes that displayed a comparable lack of branch support for the backbone of the phylogenetic tree (Song et al. 2012). In terms of nucleotide composition, the *C. lectularius* mitogenome was slightly purine-rich (52%) with a high AT content (71%). Compositional bias was pronounced (Figure 1(B)), as is common in insects (Cameron 2014) with high AT bias across tRNA loci and third codon positions – a characteristic also shared by other Hemiptera (Liu & Liang 2013). This sequence will serve as a resource for evolutionary and comparative genomics studies of true bugs and other animals, as well as the basis of microevolutionary studies of bed bug colonization, infestation, and heteroplasmy (Robison et al. 2015).

**Figure 1.**
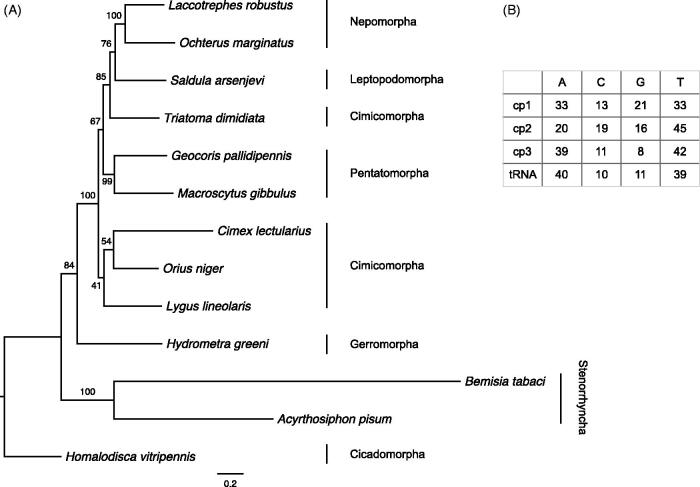
(A) Phylogenetic relationships among Hemiptera species based on the translated sequences of 13 protein-coding genes. Values at the internode branches denote support drawn from 500 rapid bootstrap pseudoreplicates (Stamatakis et al. 2008) mapped onto the best-known maximum likelihood phylogram (−ln Lik =63126.175, *α* = 0.53). Infraorder groupings are indicated. GenBank accessions used: *Acyrthosiphon pisum* (NC_011594), *Bemicia tabaci* (NC_006279), *Geocoris pallidipennis* (NC_012424), *Homalodisca vitripennis* (NC_006899), *Hydrometra greeni* (NC_012842), *Laccotrephes robustus* (NC_012817), *Lygus lineolaris* (NC_021975), *Macroscytus gibbulus* (NC_012457), *Ochterus marginatus* (NC_12820), *Orius niger* (NC_12429), *Saldula arsenjevi* (NC_012463), *Triatoma dimidiata* (NC_002609). (B) Nucleotide composition for each codon position and all tRNA loci.
