# Erratum

**DOI:** 10.1080/23802359.2016.1224663

**Published:** 2016-09-06

Kolokotronis S-O, Foox J, Rosenfeld JA, Brugler MR, Reeves D, Benoit JB, Booth W, Robison G, Steffen M, Sakas Z, Palli SR, Schal C, Richards S, Narechania A, Baker RH, Sorkin LN, Amato G, Mason CE, Siddall ME, DeSalle R. (2016). The mitogenome of the bed bug *Cimex lectularius* (Hemiptera: Cimicidae). [epub ahead of print]

http://dx.doi.org/10.1080/23802359.2016.1180268

When the above article was first published online, author ‘Zoe Sakas’ was listed at the end in the author group. And on the first page of the article, two scientific names - *Orius niger* and *Lygus lineolaris* were not italicized.

In the corrected version of the article, name of the author ‘Zoe Sakas’ is placed after ‘Michael Steffen’ and the scientific names are italicized.

Taylor & Francis apologises for this error.

